# Increased Levels of Oxidative Stress Markers, Soluble CD40 Ligand, and Carotid Intima-Media Thickness Reflect Acceleration of Atherosclerosis in Male Patients with Ankylosing Spondylitis in Active Phase and without the Classical Cardiovascular Risk Factors

**DOI:** 10.1155/2017/9712536

**Published:** 2017-08-15

**Authors:** Agata Stanek, Armand Cholewka, Tomasz Wielkoszyński, Ewa Romuk, Karolina Sieroń, Aleksander Sieroń

**Affiliations:** ^1^School of Medicine with the Division of Dentistry in Zabrze, Department of Internal Medicine, Angiology and Physical Medicine, Medical University of Silesia, Batorego St., 15, 41-902 Bytom, Poland; ^2^Department of Medical Physics, Chelkowski Institute of Physics, University of Silesia, 4 Uniwersytecka St., 40-007 Katowice, Poland; ^3^School of Medicine with the Division of Dentistry in Zabrze, Department of Biochemistry, Medical University of Silesia, Jordana 19 St., 41-808 Zabrze, Poland; ^4^School of Health Sciences in Katowice, Department of Physical Medicine, Chair of Physiotherapy, Medical University of Silesia, Medyków St., 12, 40-752 Katowice, Poland

## Abstract

**Objective:**

The primary aim of the study was to assess levels of oxidative stress markers, soluble CD40 ligand (sCD40L), serum pregnancy-associated plasma protein-A (PAPP-A), and placental growth factor (PlGF) as well as carotid intima-media thickness (IMT) in patients with ankylosing spondylitis (AS) with active phase without concomitant classical cardiovascular risk factors.

**Material and methods:**

The observational study involved 96 male subjects: 48 AS patients and 48 healthy ones, who did not differ significantly regarding age, BMI, comorbid disorders, and distribution of classical cardiovascular risk factors. In both groups, we estimated levels of oxidative stress markers, lipid profile, and inflammation parameters as well as sCD40L, serum PAPP-A, and PlGF. In addition, we estimated carotid IMT in each subject.

**Results:**

The study showed that markers of oxidative stress, lipid profile, and inflammation, as well as sCD40L, PlGF, and IMT, were significantly higher in the AS group compared to the healthy group.

**Conclusion:**

Our results demonstrate that ankylosing spondylitis may be associated with increased risk for atherosclerosis.

## 1. Introduction

Ankylosing spondylitis (AS) is a chronic inflammatory arthritis affecting primarily the axial skeleton and sacroiliac joints [1, 2].

Several epidemiological studies confirmed the high risk of cardiovascular morbidity and mortality in AS patients, which is associated with an increase of risk for atherosclerosis independent of traditional risk factors that may be connected with the disease activity, functional and mobility limitations, structural damage, and inflammation [3, 4].

Inflammation, on the one hand, has an important role in different stages of atherogenesis, and, on the other, attenuates established cardiovascular risk factors [5, 6].

During inflammation and phagocytosis reactions, reactive oxygen species (ROS) may be released into the extracellular space and injure the surrounding tissue and thereby result in the production of acute-phase proteins [7].

Moreover, according to the theory of oxidative stress, atherosclerosis results from the oxidative modification of low-density lipoproteins (LDL) in the arterial wall by ROS [8]. Modified ox-LDL is generally accepted to be an important elicitor of promitotic, proinflammatory, and atherogenic effects in vascular cells [9]. What is more, ox-LDL may interact with different molecules and form proatherogenic complexes (e.g., ox-LDL/CRP and ox-LDL/*β*2-glycoprotein 1) that may not only perpetuate vascular inflammation but also trigger autoimmune responses, accelerating the development of atherosclerosis [10, 11].

In addition, high titers of antibodies of ox-LDL have been reported in patients with myocardial infarction [12], atheroslerosis [13], and rheumatoid disease [9].

It has been demonstrated that the CD40L concentration is increased in patients with occlusive carotid artery disease and may be also a predictor of cardiovascular events [14, 15].

On the other hand, it has been also reported that the placental growth factor (PlGF) plays an important role in atherosclerosis by stimulating the angiogenesis and atherogenic migration of monocytes/macrophages into the arterial wall [16]. It seems to be more effective during the early phase of atherogenesis, because anti-PlGF antibody treatment significantly inhibits early lesions but is ineffective during the more advanced stages of plaque development [17].

The other marker of atherogenesis is serum pregnancy-associated plasma protein-A (PAPP-A). It has been shown that its circulating concentrations are higher in patients with acute coronary syndrome than in patients with chronic stable angina and in healthy subjects. Furthermore, increased serum PAPP-A concentration is associated with the presence and extent of stable coronary heart disease as well as predictive of future ischemic cardiac events, and the need for percutaneous coronary intervention or coronary artery bypass graft surgery [18, 19].

On the other hand, a few papers postulate that oxidative stress might be involved both in AS disease onset and progression [20–24].

Taking this data into account, the primary aim of the study was to assess levels of oxidative stress markers, sCD40L, PAPP-A, and PlGF as well as carotid IMT in AS patients with active phase without concomitant classical cardiovascular risk factors.

## 2. Materials and Methods

### 2.1. Participants

The research protocol has been reviewed and approved by the Bioethical Committee of the Medical University of Silesia in Katowice (Permission number KNW/022/KB/103/16), and all the subjects we analyzed gave their informed, written consent for inclusion in the observational study. It was carried out in accordance with the Declaration of Helsinki (1964). The study involved 96 male subjects: 48 patients with ankylosing spondylitis (AS group, mean age 46.06 ± 1.44 years) and 48 healthy subjects (control group, mean age 46.63 ± 1.50 years), who did not differ significantly regarding age, BMI, comorbiding disorders, and distribution of classical cardiovascular risk factors. All the patients included in the observational study fulfilled the modified New York Criteria for definite diagnosis of AS, which served as the basis for the ASAS/EULAR recommendations [25]. Enrollment in the study was performed in the AS group of male patients, with definite diagnosis of AS who did not suffer from any other diseases, had no associated pathologies, and whose attending physician did not apply disease-modifying antirheumatic drugs (DMARDs), biologic agents, or steroids. The AS patients were treated with NSAIDs, which doses were not altered within one month before the beginning of the study. All the patients with AS were HLAB27 positive, and they exhibited III and IV radiographic grades of sacroiliac joint disease. The BASDAI was 5.35 ± 1.64 and the BASFI was 5.13 ± 2.17. The AS patients did not suffer from any other diseases. Similarly, the healthy subjects had no acute or chronic diseases, nor did they use any medication. The demographic data of the subjects is shown in Table 1.

The subjects from both groups were asked to abstain from alcohol, drugs, and any immunomodulators, immunostimulators, hormones, vitamins, minerals, or other substances with antioxidant properties for 4 weeks before the study. All the subjects were also asked to refrain from the consumption of caffeine 12 hours prior to laboratory analysis.

### 2.2. Blood Sample Collection

Blood samples of all the subjects were collected in the morning before the first meal. Samples of whole blood (5 ml) were drawn from a basilic vein of each subject and then collected into tubes containing ethylenediaminetetraacetic acid (Sarstedt, S-monovette with 1.6 mg/ml EDTA-K_3;_ catalogue number 04.1931) and into tubes with a clot activator (Sarstedt, S-monovette, catalogue number 04.1934). The blood samples were centrifuged (10 min., 900*g*, 4°C), and then the plasma and serum were immediately separated and stored at the temperature of −70°C, until biochemical analyses were performed. In turn, the red blood cells retained from removal of EDTA-plasma were rinsed with isotonic salt solution and then 10% of hemolysates were prepared for further analyses. Hemoglobin concentration in hemolysates was determined by standard cyanmethemoglobin method. The inter- and intra-assay coefficients of variations (CV) were, respectively, 1.1% and 2.4%.

### 2.3. Biochemical Analyses

#### 2.3.1. Oxidative Stress Marker Analyses

*(1) Determination of Activity of Antioxidant Enzymes*. The plasma and erythrocyte superoxide dismutase (SOD—E.C.1.15.1.1) activity was determined by the Oyanagui method [26]. Enzymatic activity was expressed in nitrite unit (NU) in each mg of hemoglobin (Hb) or ml of blood plasma. One nitrite unit (1 NU) means a 50% inhibition of nitrite ion production by SOD in this method. SOD isoenzymes (SOD-Mn and SOD-ZnCu) were measured using potassium cyanide as the inhibitor of the SOD-ZnCu isoenzyme. The inter- and intra-assay coefficients of variations (CV) were, respectively, 2.8% and 5.4%.

The catalase (CAT—E.C.1.11.1.6.) activity in erythrocytes was measured by Aebi [27] kinetic method and expressed in IU/mgHb. The inter- and intra-assay coefficients of variations (CV) were, respectively, 2.6% and 6.1%.

The erythrocyte glutathione peroxidase (GPx—E.C.1.11.1.9.) activity was assayed by Paglia and Valentine's kinetic method [28], with t-butyl peroxide as a substrate and expressed as micromoles of NADPH oxidized per minute and normalized to one gram of hemoglobin (IU/gHb). The inter- and intra-assay coefficients of variations (CV) were, respectively, 3.4% and 7.5%.

The activity of glutathione reductase in erytrocytes (GR—E.C.1.6.4.2) was assayed by Richterich's kinetic method [29], expressed as micromoles of NADPH utilized per minute and normalized to one gram of hemoglobin (IU/gHb). The inter- and intra-assay coefficients of variations (CV) were, respectively, 2.1% and 5.8%.

*(2) Determination of Nonenzymatic Antioxidant Status*. The total antioxidant capacity of plasma was measured as the ferric reducing ability of plasma (FRAP) according to Benzie and Strain [30] and calibrated with the use of Trolox and expressed in *μ*mol/l. The inter- and intra-assay coefficients of variations (CV) were, respectively, 1.1% and 3.8%.

The serum concentration of protein sulfhydryl (PSH) was determined by Koster et. al's method [31] using dithionitrobenzoic acid (DTNB) and expressed in *μ*mol/l. The inter- and intra-assay coefficients of variations (CV) were, respectively, 2.6% and 5.4%.

The serum concentration of uric acid (UA) was determined by a uricase-peroxidase method [32] on the Cobas Integra 400 plus analyzer and expressed as mg/dl. The inter- and intra-assay coefficients of variations (CV) were, respectively, 1.4% and 4.4%.

*(3) Determination of Lipid Peroxidation Products and TOS*. The intensity of lipid peroxidation in the plasma and the erythrocytes was measured spectrofluorimetrically as a thiobarbituric acid-reactive substances (TBARS) according to Ohkawa et al. [33]. The TBARS concentrations were expressed as malondialdehyde (MDA) equivalents in *μ*mol/l in plasma or nmol/gHb in erythrocytes. The inter- and intra-assay coefficients of variations (CV) were, respectively, 2.1% and 8.3%.

The serum concentrations of oxidized low-density lipoprotein (ox-LDL) and antibodies to ox-LDL (ab-ox-LDL) were measured with the use of ELISA kits (catalogue numbers BI-20022 and BI-20032, Biomedica, Poland) according to the manufacturer's instructions. The ox-LDL and the ab-ox-LDL concentrations were expressed in ng/ml and mU/ml, respectively. The inter- and intra-assay coefficients of variations (CV) for ox-LDL were 5.8% and 9.4%, respectively, and for ab-ox-LDL—4.1% and 8.7%, respectively.

The serum total oxidant status (TOS) was determined with the method described by Erel [34] and expressed in *μ*mol/l. The inter- and intra-assay coefficients of variations (CV) were, respectively, 2.2% and 6.4%.

#### 2.3.2. Determination of Inflammatory State Parameters

The erythrocyte sedimentation rate (ESR) was determined immediately in whole blood with EDTA by the classical Westergren method.

The high-sensitivity C-reactive protein (hs-CRP) (catalogue number EIA 4584) concentration in serum was determined by latex immunoturbidimetric method (BioSystems, Spain) and expressed in mg/l. The inter- and intra-assay coefficients of variations (CV) were, respectively, 2.3% and 5.5%.

The serum ceruloplasmin (CER) oxidase activity was measured with the use of the p-phenylenediamine kinetic method by Richterich [29] and expressed in mg/dl after a calibration with pure ceruloplasmin isolated from a healthy donor serum pool. The inter- and intra-assay coefficients of variations (CV) were, respectively, 3.1% and 6.1%.

#### 2.3.3. Determination of Lipid Profile

Total, HDL-, and LDL-cholesterol (T-Chol, HDL-Chol, and LDL-Chol, resp.) and triglicerydes (TG) concentrations in serum were estimated using routine techniques (Cobas Integra 400 plus analyzer, Roche Diagnostics, Mannheim, Germany). The concentrations were expressed in mg/dl. The inter- and intra-assay coefficients of variations (CV) were, respectively, 2.8% and 5.4% for T-Chol, 3.2% and 5.4% for HDL-Chol, 2.6% and 6.5% for LDL-Chol, and 2.5% and 7.6% for TG.

The triglycerides/HDL-cholesterol (TG/HDL) and LDL-cholesterol/HDL-cholesterol (LDL/HDL) ratios were calculated.

#### 2.3.4. Determination of PAPP-A, Soluble CD40 Ligand, and PlGF

Serum pregnancy-associated plasma protein-A (PAPP-A) (catalogue number EIA-4512), soluble CD40 Ligand (sCD40L) (catalogue number EIA4851), and placental growth factor (PlGF) (catalogue number EIA-4529) concentrations were assayed by ELISA methods with DRG Instruments GmbH (Germany) kits. All assays were performed according to the manufacturer's instructions. The PAPP-A and sCD40L concentrations were expressed in ng/ml, the PlGF concentration—in pg/ml. The inter- and intra-assay coefficients of variations (CV) were, respectively, 6.8% and 10.2% for PAPPA-A, 5.1% and 9.4% for sCD40L, and 6.2% and 12.1% for PlGF.

### 2.4. Assay of Intima-Media Thickness

A high-resolution Doppler ultrasonography was performed with a Logic-5 device with a high-frequency (11 MHz and 15 MHz) linear probe. The sonographer was an angiologist who was unaware of subject's clinical state. Measurement of intima-media thickness (IMT) was performed in the right and left common carotid arteries, and the average of the 2 measurements was calculated. The IMT was expressed in mm.

### 2.5. Assay of Activity of Ankylosing Spondylitis

The activity of ankylosing spondylitis was measured by the Bath Ankylosing Spondylitis Disease Activity Index (BASDAI) and the Bath Ankylosing Spondylitis Functional Index (BASFI).

The BASDAI has six questions related to fatigue, back pain, peripheral pain, peripheral swelling, local tenderness, and morning stiffness (degree and length). Other than the item relating to morning stiffness, all questions were scored from 0 (none) to 10 (very severe) using a visual analogue scale (VAS). The sum was calculated as the mean of two morning stiffness items and the four remaining items [35].

The BASFI is the mean score of ten questions addressing functional limitations and the level of physical activity at home and work, assessed on VAS scales (0 = easy, 10 = impossible) [36].

### 2.6. Statistical Analyses

Statistical analyses were undertaken using the statistical package of Statistica 10 Pl software. For each parameter, the indicators of the descriptive statistics were determined (mean value and standard deviation (SD)). The normality of the data distribution was checked using the Shapiro-Wilk test, while the homogeneity of the variance was checked by applying the Levene test. In order to compare the differences between the control group and the AS group, an independent sample Student's *t*-test was used or alternatively the Mann–Whitney *U* test. Correlations between particular parameters were statistically verified by means of Spearman's nonparametric correlation test. Differences at the significant level of *p* < 0.05 were considered statistically significant.

## 3. Results

### 3.1. Oxidative Stress

In AS patients, there was a significantly higher activity of antioxidant enzymes: plasma SOD, along with erythrocyte SOD, erythrocyte CAT, erythrocyte GPx, and erythrocyte GR, was observed in comparison to the healthy subjects. But the plasma activity of SOD-Mn and SOD-CuZn isoenzymes in both groups did not differ significantly. What is more, in AS patients, a significantly lower concentration of the parameters of nonenzymatic antioxidants, FRAP, PSH, and UA, was observed in comparison to the healthy subjects (Table 2).

Furthermore, a significantly higher concentration of lipid peroxidation products plasma MDA, along with erythrocyte MDA, ox-LDL, and ab-ox-LDL, was noted in AS patients in comparison to the control group of the healthy subjects. In addition, in AS patients, the concentration of TOS was significantly higher in comparison to the control group (Table 3).

### 3.2. Lipid Profile, Inflammatory Parameters, and Carotid Intima-Media Thickness

In AS patients, there was a significantly higher concentration of the lipid profile parameters: T-Chol, LDL-Chol, and TG as well as TG/HDL ratio and LDL/HDL ratio were noted in comparison to the control group. But only the concentration of HDL-Chol in both groups did not differ significantly. What is more, also the concentrations of sCD40L and PlGF as well as carotid IMT were significantly higher in AS patients in comparison to the healthy group. The concentration of PAPP-A in both groups did not differ significantly. Furthermore, in AS patients, a significantly higher concentration of all the examined parameters of inflammatory state, ESR, hs-CRP, and CER, was observed in comparison to the healthy subjects (Table 4).

### 3.3. Significant Relationships among the Estimated Parameters in AS Patients

In the AS group, a high, statistically significant correlation was observed between acute phase proteins (hs-CRP versus CER; *r* = 0.59, *p* < 0.05), total SOD activity in erythrocytes and ceruloplasmin (*r* = 0.52, *p* < 0.05), and glutathione cycle enzyme activities in erythrocytes (POX versus GR; *r* = 0.66, *p* < 0.05) as well as between uric acid concentration and FRAP activity in plasma (*r* = 0.65, *p* < 0.05). Mild but still statistically significant correlations were shown between CRP concentration and SOD-CuZn and SOD-Mn plasma activities (*r* = −0.35, *p* < 0.05), erythrocyte MDA concentration and SOD erythrocyte activity (*r* = 0.35, *p* < 0.05), sCD40L and PlGF concentration (*r* = 0.51, *p* < 0.05), plasma MDA and T-Chol (*r* = 0.30, *p* < 0.05), and TOS and TG/HDL ratio (*r* = 0.37, *p* = 0.001). Also, a high correlation between BASFI and BASDAI was observed (*r* = 0.67, *p* < 0.05) in AS patients.

Unfortunately, no statistically significant correlations between AS activity parameters (BASFI and BASDAI) and oxidative stress parameters as well as carotid IMT and oxidative stress parameters were obtained.

## 4. Discussion

Briefly, in this observational study, we viewed significantly higher oxidative stress parameters, levels of inflammatory state, and lipid profile parameters, sCD40L, and PlGF as well as values of TG/HDL, LDL/HDL ratio, and carotid IMT in AS patients with active phase (BASDAI and BASFI), compared to the healthy subjects.

In the available literature, only a few, unequivocal reports concerning the prooxidant-antioxidant status in patients with ankylosing spondylitis have been published.

In the study [20], in patients with ankylosing spondylitis, a significantly lower plasma total antioxidant status (TAS) was demonstrated, as well as higher values of the total oxidant status (TOS) and oxidative stress index (OSI), in comparison to the control group of healthy volunteers. That study did not reveal any significant correlation between the values of the above parameters and the activity of the disease process.

In another study [22], no significant differences were demonstrated to occur in the activity of SOD, nitric oxide (NO) metabolites, and the concentration of MDA, between the group of patients with AS in the active form and the group of patients with inactive process. The activity of SOD, NO metabolites, and the concentration of MDA also failed to demonstrate statistically significant differences when compared to the control group of healthy subjects, whereas the activity of CAT and the concentration of MDA in patients with an active form of the disease were significantly higher, in relation to other groups of subjects studied.

In the study [37], all antioxidant enzyme activities were lower, but the MDA level was higher in patients with AS when compared to the control group.

The next paper [38] reported that ESR, CRP, and lipid peroxidation products were higher in patients with AS than in healthy subjects, but vitamins A, C, E, and *β*-carotene concentrations in plasma, reduced glutathione, and glutathione peroxidase activity values in erythrocyte were lower in patients with AS than in healthy subjects. But the authors estimated only some chosen parameters of prooxidant-antioxidant status. Furthermore, the level of oxidative stress was shown to be correlated with the intensity of inflammation in patients with AS [21].

Contrastingly, there are many papers, which reported increased cardiovascular risk in AS patients [39–43].

In the current study, a significant increase in acute-phase protein concentration (hs-CRP and CER) was observed in AS patients compared to the healthy subjects. A significant positive correlation between hs-CRP and CER was shown as well as a significant negative correlation between hs-CRP and plasma SOD-CuZn.

Some researchers found that increased AS disease activity was associated with decreases in lipid levels and the decrease in HDL-Chol levels, which tended to be almost twice as large as the decrease in total cholesterol levels, resulting in a more atherogenic lipid profile [43, 44].

However, in the study [45] in AS patients, the authors did not observe a significant difference in T-Chol, LDL-Chol, HDL-Chol, and TG concentration compared to healthy subjects. But in AS patients, the values of HDL/LDL ratio, complex intima-media, and TOS concentration as well as inflammatory state parameters were significantly higher than in controls. In this study, a significant increase in T-Chol, LDL-Chol, and TG as well as TG/HDL and LDL/HDL ratio was observed in comparison to the healthy subjects. But HDL-Chol concentration did not differ between AS patients and healthy subjects. In addition, the carotid IMT in AS patients was also significantly higher in comparison to the healthy subjects.

A high level of cholesterol, especially in LDL, may activate thrombocytes and cause the release of substances that activate phospholipase A_2_. Then, the accumulated arachidonic acid is metabolized to leukotriene by a lipoxygenase pathway and thromboxane, prostaglandin, and MDA by a cyclooxygenase pathway. During this metabolism, ROS may be produced, and under insufficient antioxidant capacity, they may also trigger lipid peroxidation [44]. What is more, it was shown that the TG/HDL ratio estimates atherogenic small, dense low-density lipoprotein cholesterol and predicts arterial stiffness and hard cardiovascular events in adults [46, 47]. In our study, we observed a significant positive correlation between TOS and TG/HDL ratio.

Additionally, it was also shown that an increase in serum LDL levels leads to an increase in the adherence of circulating monocytes to arterial endothelial cells and at the same time to an increased rate of entry of LDL into the intima [48]. It is also possible that TG enrichment may alter the physicochemical properties of LDL, which is considerably more susceptible to oxidation [49].

In this study, we also observed the increased concentration of lipid peroxidation products in plasma and erythrocyte.

MDA is one the most abundant aldehydes, resulting from peroxidation of arachidonic, eicosapentaenoic, and docosahexaenoic acid [50, 51]. MDA reacts with lysine residues by forming Schiff bases [52] and plays a major role in LDL modification and their deviation towards macrophages [48].

In the current study, a significantly higher concentration of MDA in plasma as well as in erythrocytes in AS patients was observed compared to healthy subjects. A positive correlation between plasma MDA and T-Chol as well as erythrocyte MDA and SOD in AS patients was also shown. We did not observe any correlation between MDA and other estimated parameters. The explanation of this fact may be that TBARS assay does not measure MDA exclusively, because it reacts to compounds other than MDA [51].

However, so far, there are no reports estimating ox-LDL and ab-ox-LDL concentration in ankylosing spondylitis. In the present study, a significantly higher concentration of ox-LDL as well as ab ox-LDL in AS patients was observed compared to healthy subjects.

In the study [53], it was shown that antibodies to ox-LDL were correlated significantly with ESR and CRP in patients with early rheumatoid arthritis and suggested that the occurrence of these antibodies must be related to inflammation. However, in the current study, no correlation was observed between ox-LDL and ab-ox-LDL and other estimated parameters.

In our study, we observed a significantly higher levels of sCD40L and PlGF as well as a positive correlation between them. Furthermore, in this study, the level of PAPP-A did not differ between AS patients and healthy subjects. It may be connected with the fact that PAPP-A is expressed in unstable but not in stable atherosclerotic plaques [18].

The study proved that increased oxidative stress, the levels of sCD40L and PlGF, the disturbance of lipids, and the inflammation process may enhance atherogenesis in AS patients.

However, the study has some limitations. First, it involved only 48 AS patients and thus a greater number of patients should be examined. Second, patients in different stages of AS should be involved in the study.

## 5. General Conclusion

Our results demonstrate that increased oxidative stress, higher serum concentrations of PlGF and sCD40L, and increased IMT may reflect the acceleration of atherosclerosis in male AS patients in active phase and without concomitant classical cardiovascular risk factors.

## Figures and Tables

**Table 1:** Demographic data of the study subjects.

Characteristic	AS patients (*n* = 48)	Healthy subjects (*n* = 48)	*p* value
Sex (M/F)	48/0	48/0	—
Age (years), mean (SD)	46.06 ± 1.44	46.63 ± 1.50	0.096
BMI (kg/m^2^), mean (SD)	24.5 ± 4.4	23.8 ± 5.7	0.674
Smoking (yes/no)	0/48	0/48	—
BASDAI	5.35 ± 1.64	—	—
BASFI	5.13 ± 2.17	—	—
HLA B27 antigen (yes/no)	48/0	—	—
Cerebral/coronary/peripheral vascular disease (yes/no)	0/48	0/48	—
Hypertension (yes/no)	0/48	0/48	—
Diabetes mellitus (yes/no)	0/48	0/48	—
Hyperlipidemia (yes/no)	0/48	0/48	—
Medication			
NSAID (yes/no)	48/0	—	—
DMARD (yes/no)	0/48	—	—
Biological agents (yes/no)	0/48	—	—

SD: standard deviation; BMI: body mass index; BASDAI: the Bath Ankylosing Spondylitis Disease Activity Index; BASFI: the Bath Ankylosing Spondylitis Functional Index; HLA B27 antigen: human leukocyte B27 antigen; NSAID: nonsteroidal anti-inflammatory drug; DMARD: disease-modifying antirheumatic drug.

**Table 2:** Parameters of enzymatic antioxidant status (superoxide dismutase (SOD), its isoenzymes: manganese superoxide dismutase (SOD-Mn) and copper-zinc superoxide dismutase (SOD-CuZn), catalase (CAT), glutathione peroxidase (GPx), and glutathione reductase (GR) activity) and nonenzymatic antioxidant status (ferric reducing ability of plasma (FRAP), protein sulfhydryl (PSH), and uric acid (UA) concentration, as well as activity of ceruloplasmin (CER)) in ankylosing spondylitis (AS) patients and healthy subjects.

Parameter	AS patients (*n* = 48)	Healthy subjects (*n* = 48)	*p*
SOD (p) (NU/ml)	12.67 ± 1.98	10.93 ± 2.55	**<0.001**
SOD-Mn (p) (NU/ml)	5.08 ± 2.00	4.59 ± 1.88	0.223
SOD-CuZn (p) (NU/ml)	7.64 ± 2.31	6.79 ± 1.99	0.055
SOD (e) (NU/mgHb)	105.85 ± 22.60	95.50 ± 19.18	**0.017**
CAT (e) (IU/mgHb)	410.98 ± 63.56	352.55 ± 77.21	**<0.001**
GPx (e) (IU/gHb)	27.23 ± 6.43	24.49 ± 5.00	**0.022**
GR (e) (IU/gHb)	1.67 ± 0.58	1.38 ± 0.43	**0.007**
FRAP (p) (*μ*mol/l)	550.38 ± 76.98	642.17 ± 105.67	**<0.001**
PSH (s) (*μ*mol/l)	474.46 ± 192.06	559.07 ± 215.14	**0.045**
UA (s) (mg/dl)	4.73 ± 1.39	5.84 ± 1.53	**<0.001**

Values are expressed as means ± standard deviations (SD) of the means; p: plasma; s: serum; e: erythrocyte lysates.

**Table 3:** Oxidative stress parameters: malondialdehyde (MDA), oxidized low-density lipoprotein (ox-LDL), antibodies to oxidized low-density lipoprotein (ab-ox-LDL), and total oxidant status (TOS) concentration in ankylosing spondylitis (AS) patients and healthy subjects.

Parameter	AS patients (*n* = 48)	Healthy subjects (*n* = 48)	*p*
MDA (p) (*μ*mol/l)	2.51 ± 0.63	2.25 ± 0.47	**0.025**
MDA (e) (nmol/gHb)	0.17 ± 0.03	0.15 ± 0.03	**<0.001**
ox-LDL (s) (ng/ml)	268.48 ± 105.83	162.98 ± 63.29	**<0.001**
ab-ox-LDL (s) (mU/ml)	479.82 ± 328.39	323.82 ± 210.26	**0.007**
TOS (s) (*μ*mol/l)	26.99 ± 10.65	16.50 ± 6.87	**<0.001**

Values are expressed as means ± standard deviations (SD) of the means; p: plasma; s: serum; e: erythrocyte lysates.

**Table 4:** Parameters of lipid profile (total cholesterol (T-Chol), low-density lipoprotein cholesterol (LDL-Chol), high-density lipoprotein cholesterol (HDL-Chol), triglicerydes (TG) concentration, TG/HDL, and LDL/HDL ratio), concentration of PAPP-A, soluble CD40 ligand (sCD40L), PlGF, and value of carotid intima-media thickness (IMT), as well as parameters of inflammatory state (erythrocyte sedimentation rate (ESR) value, high sensitivity C-reactive protein (hs-CRP), and ceruloplasmin (CER) concentration) in ankylosing spondylitis (AS) patients and healthy subjects.

Parameter	AS patients (*n* = 48)	Healthy subjects (*n* = 48)	*p*
T-Chol (s) (mg/dl)	217.73 ± 35.48	187.09 ± 18.57	**<0.001**
LDL-Chol (s) (mg/dl)	140.49 ± 33.64	112.57 ± 22.89	**<0.001**
HDL-Chol (s) (mg/dl)	61.10 ± 18.08	57.49 ± 15.16	0.291
TG (s) (mg/dl)	190.48 ± 47.30	139.74 ± 47.66	**<0.001**
TG/HDL ratio	3.37 ± 1.13	2.57 ± 1.23	**<0.001**
LDL/HDL ratio	2.55 ± 1.1	2.02 ± 0.58	**<0.05**
PAPP-A (s) (ng/ml)	17.82 ± 16.22	14.24 ± 4.35	0.281
sCD40L (s) (ng/ml)	8.93 ± 3.74	5.54 ± 2.37	**<0.001**
PlGF (s) (pg/ml)	25.8 ± 8.99	19.77 ± 3.27	**<0.001**
Carotid IMT (mm)	1.1 ± 0.13	0.55 ± 0.08	**<0.001**
ESR	27.13 ± 21.55	5.94 ± 3.91	**<0.01**
hs-CRP (s) (mg/l)	14.92 ± 15.55	1.58 ± 2.00	**<0.001**
CER (s) (mg/dl)	48.12 ± 12.67	38.68 ± 4.84	**<0.001**

Values are expressed as means ± standard deviations (SD) of the means; s: serum.
